# Behavioural responses of spinner dolphins to human interactions

**DOI:** 10.1098/rsos.172044

**Published:** 2018-04-25

**Authors:** Maddalena Fumagalli, Amina Cesario, Marina Costa, John Harraway, Giuseppe Notarbartolo di Sciara, Elisabeth Slooten

**Affiliations:** 1 Department of Zoology, University of Otago, 340 Great King Street, Dunedin 9054, New Zealand; 2 Tethys Research Institute, Viale G.B. Gadio 2, Milano 20121, Italy; 3 The Swire Institute of Marine Science, University of Hong Kong, Pokfulam Road, Hong Kong SAR, People's Republic of China; 4 South Atlantic Environmental Research Institute (SAERI), Stanley Cottage, Falkland Islands; 5 Department of Mathematics and Statistics, University of Otago, 730 Cumberland Street, Dunedin 9016, New Zealand

**Keywords:** behavioural response, tourism impact, dolphin conservation, spinner dolphin, log-linear analysis, stationary state distribution

## Abstract

There is increasing evidence that whale and dolphin watching activities have detrimental effects on targeted cetacean populations. In Egypt, spinner dolphins regularly occur in the resting areas of Samadai, Satayah and Qubbat'Isa reefs. In-water human interactions with dolphins are regulated with a time-area closure system at Samadai, unregulated at Satayah and non-existent at Qubbat'Isa. This provided an ideal experimental setting to advance our understanding of the effects of tourism on a species highly sensitive to disturbances. Our study confirmed that the intensity and duration of interactions, and therefore, dolphin exposure to tourism, differed among the study sites. Compared with the Qubbat'Isa control site, behavioural reactions to boats and swimmers at the two tourism sites suggested that dolphin rest was disrupted, especially around the middle of the day and especially at Satayah, where dolphin tourism is unregulated. Our results indicate also that the dolphin protection measures at Samadai reduce the level of disturbance. We recommend that similar measures be implemented at other dolphin tourism locations, and that no new operations be initiated until the long-term impacts on dolphin populations are better understood. Our experience emphasizes the need to adopt precautionary approaches in research and management of whale and dolphin watching.

## 1. Introduction

Commercial whale watching encompasses a range of tourism operations intended for profit, in which paying participants seek interactions with wild cetaceans (whales, dolphins and porpoises) [1]. The industry is on the rise globally [2], with expected further growth [3], and it is often managed in ways that are neither ecologically nor socially sustainable [4]. The dominant narrative has long presented whale watching as an activity supporting cetacean conservation [5]. However, there is growing evidence that behaviour change can occur in the animals targeted [6], and that this industry is potentially sub-lethal and consumptive in nature [7]. Concern for the conservation of targeted marine mammal populations has led the UN Convention on Migratory Species of Wild Animals to adopt at its 2017 Conference of Parties a resolution calling for the implementation of measures such as ‘national guidelines, codes of conduct, and if necessary, national legislation, binding regulations or other regulatory tools, to address the consequences of, and carefully regulate, recreational in-water interactions with aquatic mammals’ [8].

Scientific investigation of the impacts of tourism on targeted cetacean species is a field of research that poses many challenges. Impact assessment is complicated by the fact that the tourism industry repeatedly seeks specific individuals or populations, hence causing effects of disturbances that are cumulative, rather than catastrophic, and can manifest at variable spatial and temporal distances from the source of impact [9,10]. Another common and serious limitation is the lack of baseline data on species behaviour, abundance and distribution under pre-tourism or control conditions [9,11]. This prevents the application of study designs aimed to compare behaviour under control and disturbed conditions (e.g. Before–During–After, Before–After–Control–Impact [9,12]) that could indicate the significance of impacts [13] and help define quantitative criteria for sustainable management of the industry [4].

Despite the challenges, behavioural studies have shown that cetaceans respond to tourism disturbance [6] and that there is natural variability in the responses adopted by different species [6] and individuals [14]. In the majority of species investigated, rest is the behavioural state most sensitive to disruptions [6]. It is also a critical state, as reductions and interruptions of resting periods can negatively affect other behaviours of survival value (communication, navigation, foraging) and complex tasks [15], ultimately resulting in decreased survival and reproductive success of individuals and populations [16]. Animals can respond to disturbances by adjusting their behaviour and compensating for the lost rest at other times, or locations, when possible. Species and individuals for which one or more of the survival functions are constrained to specific times or locations, however, would be less able to successfully rearrange their behavioural budget, and are anticipated to be less resilient to disruptions [17].

The spinner dolphin (*Stenella longirostris*) is considered particularly vulnerable to human pressures [18,19]. The typical behaviour pattern of the species is to feed at night [20] and rest during the day [21]. Across its distribution range, some populations rest within the confines of bays, inlets and lagoons [19,21]. These resting areas share common environmental features (e.g. sandy substrate, shallow depth [21,22]) that make them safe havens from predators, maximizing survival during a phase of decreased vigilance. Schools enter the resting areas at dawn and leave before dusk [19,21], displaying a clear phase of rest in the morning [21] or midday hours [19]. This state is easily recognized by the predictable, coordinated, synchronous slow swimming of tight groups, lack of aerial displays, coordinated breathing patterns with short surface intervals and very little acoustic communication [21]. The accessibility and the predictable occurrence of dolphin schools have favoured the establishment of dolphin watching and swim-with operations in spinner dolphin resting areas at several locations, including Hawai'i [21,23–25], French Polynesia [26], Indonesia [27], Fiji [28], Mauritius [29] and Egypt [30]. At some of these tourist locations, spinner dolphins have been observed to interrupt and reduce rest in the presence of tourists [23,31,32]. Long-term studies in Hawai'i have recently detected a decline in abundance since the 1990s, potentially caused by the increasing tourism pressure in the resting areas [33].

**Figure 1.**
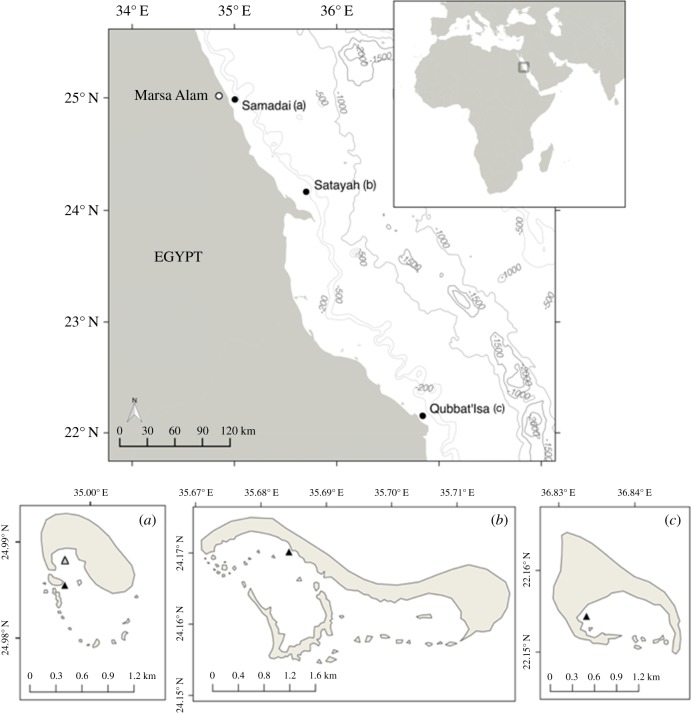
Top: Map of the Southern Egyptian Red Sea and the spinner dolphin resting areas examined (black dots). Bottom: Profile of Samadai (*a*), Satayah (*b*) and Qubbat'Isa Reef (*c*). Triangle = research vessel mooring site in 2006 (grey) and 2013–2014 (black).

In Egypt, the popularity of in-water spinner dolphin encounters has increased exponentially since the 1990s [2], mainly in the resting areas of Samadai and Satayah reefs [30]. At Samadai Reef, activities have been regulated with a time-area closure system enforced since 2003: they are allowed from 08.00 to 15.00 and only outside the confines of a dolphin-only area (Zone A), which corresponds to the innermost portion of the lagoon [30]. At Satayah Reef, access and interactions are unregulated and unrestricted. A third site, Qubbat'Isa Reef, is remote and located within a military area, hence has no tourism (figure 1). This constitutes an ideal experimental setting to investigate the characteristics of human interactions and their potential impacts on the local spinner dolphin population. In order to gain a better understanding of the species' susceptibility and response to disturbance, we compared and contrasted dolphin behaviour under both pressure and control conditions, in the reefs of Samadai, Satayah and Qubbat'Isa. Our research effort not only provides original data from this region, but also is urgently required to inform the management of operations at these locations for the protection of the species.

## 2. Material and methods

### 2.1. Fieldwork

Surveys were carried out in the Southern Egyptian Red Sea (figure 1), in the lagoons of Samadai (SM, 24.99° N, 35.00° E, figure 1*a*), Satayah (ST, 24.16° N, 35.70° E, figure 1*b*) and Qubbat'Isa reefs (QI, 22.15° N, 36.84° E, figure 1*c*), during the summer months of 2006, 2011 and 2013–2014 (table 1) as part of three related, yet distinct research projects (for more details, see electronic supplementary material, S1). All reefs reach the sea surface and create lagoons 1–20 m deep, with mainly sandy substrate, open to the south and sheltered from the predominant northerly winds [34]. Visibility in the air was always optimal (greater than 2 km) and the sea conditions inside the lagoons good (Douglas scale <3), regardless of wind speed.

**Table 1.** Summary of characteristics of tourism and fieldwork in the study sites.

			field season (year and code)			
site	tourism since	tourism regulation	2006	2011	2013	2014
Samadai	1990s	management plan	SM06		SM13	SM14
Satayah	2000s	unregulated			ST13	ST14
Qubbat'Isa	no tourism	n.a.		QI11		

### 2.2. Focal group follows

Trained observers collected data from 1 to 4 m.a.s.l., on a stationary vessel moored in the lagoon (figure 1*a–c*). At Samadai, data were collected from an inflatable vessel inside the dolphin-only area (Zone A) in 2006 and from larger motorized vessels, which required mooring in deeper areas, in other surveys (figure 1*a*). A school was defined as all dolphins inside the lagoon at any one time, and within visual range of the research team. Groups within schools were identified based on a 10 m chain rule, with dolphins 10 m or less from one another considered part of the same group [35]. If multiple groups were present, only the behaviour of the focal group was recorded. The focal group was either the only group present or the largest in sight. If group size changed during the day, the focal group was changed accordingly, and a new focal group follow started. Data collection continued as long as dolphins were in the lagoon, or the research team had to leave the site for logistical reasons.

The behavioural indicators chosen were group cohesion, aerial displays and school organization (table 2). Trained observers carried out instantaneous scans [36] at 150 s intervals to estimate school formation and focal group cohesion (table 2). If the focal group was underwater at the time of the sample, the scan was conducted at the first surfacing event, provided this occurred within 1 min from the sampling time, or the sample was left blank. This sampling regime was first employed in 2006 and was found to represent the best compromise between recording dolphin behaviour and human activities at good resolution and allowing accurate data collection and recording. It was then maintained for consistency in 2013–2014 surveys. Data from Qubbat'Isa were collected at different intervals and standardized as described in electronic supplementary material, S1. Human activities were recorded as the numbers of swimmers and boats in proximity (within approx. 300 m) to the dolphin focal group, for research and/or tourism purposes, at the time of the scan. The proximity was assessed by each observer, with the aid of an experienced researcher and using natural reference points for scale. Other activities occurring in the lagoon (e.g. snorkelling around coral reef, inflatables moving towards a dive site) but at further distance were not included in the assessment. The occurrence of aerial activity displayed by the dolphins was recorded on a continuous basis in the field, then analysed as presence or absence of aerial activity in the focal group in each sampling period (table 2). To account for natural diel patterns, the day was divided into three periods of equal length, calculated as the time elapsed since sunrise divided by the length of daylight [37]: morning (0–0.33), midday (0.34–0.66) and afternoon (0.67–1), where 0 equals sunrise and 1 equals sunset. Local sunrise and sunset times for the three sites were obtained from the Astronomical Applications Department of the US Naval Observatory (http://aa.usno.navy.mil/index.php).

**Table 2.** Definition of rest behaviour indicator states and sampling regime adopted in the focal group follows.

indicator	states	definition	sampling regime
group cohesion	tight	mode of individual distance <2 body length	instantaneous scan, 150 s
	loose	mode individual distance >2 body length	
school formation	single	all individuals in one single school	instantaneous scan, 150 s
	multiple	individuals in independent groups	
group aerial activity	inactive	no display of aerial behaviour [21]	all-occurrence sampling during the 150 s following the scan
	active	display of aerial behaviour [21]	

### 2.3. Data analysis

#### 2.3.1. Human activities

**Figure 2.**
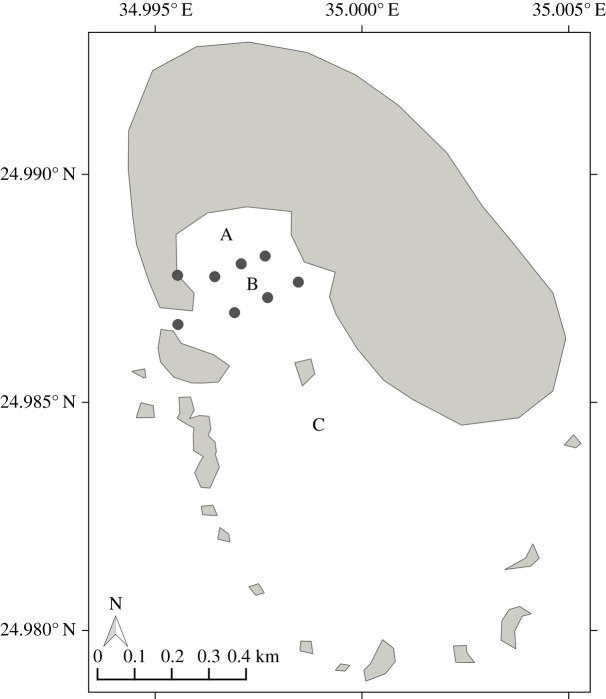
Samadai Reef zoning plan. A, dolphin-only zone; B, swimmers and snorkelers zone; C, all tourism activities and vessel morning zone.

A ‘pressure session’ is defined as series of at least five samples with human activity (for research and/or tourism purposes) never interrupted for longer than 10 min. Times of occurrence, minimum, maximum and mean duration, and the median number of boats and swimmers in sessions were calculated for each site. At Qubbat'Isa, pressure sessions involved only the inflatable boat used for photo-identification research purposes. The inflatable was equipped with an outboard engine and was manoeuvred in order to minimize disturbance to the dolphins. At Samadai, tourist interactions were only permitted between 08.00 and 15.00, whereas research activities were unrestricted and could involve two researchers swimming in Zone A to collect photo-identification data (figure 2). At Satayah Reef, as mentioned above, all human activities (i.e. dolphin interactions, diving, snorkelling) were unrestricted.

The exposure of each focal group to interactions in the morning, midday and afternoon was estimated as the proportion of samples with human activities over the total number of samples available for the focal group in the time category (e.g. if 13 of the 50 samples collected featured pressures, the exposure was 0.26, or 26%). For each full-day survey, the number of 150 s scans in which human activities were recorded provided an estimated daily cumulative duration of human activity in the vicinity of the dolphins.

#### 2.3.2. Spinner dolphin responses

Markov chains provide a robust statistical framework to assess the temporal dependence between behaviour states and the effect of predictor variables on this dependence, making them a valid tool for the assessment of tourism impacts [37–41]. This framework was adopted also in this study, which aimed to assess the persistence of states indicative of rest in the presence of human pressures, yet accounting for the circadian rhythm of the species and for differences in location and seasons. Therefore, we built chains to investigate site- and time-specific effects of disturbances on the transitions from a preceding to a succeeding behavioural state. We built control and pressure Markov chains for each field site, field season and time category. Behavioural transitions were included in the pressure chain if they occurred in the presence of human interactions. Transitions were included in the control chain when there was an absence of pressures during the sampling time and in the previous 15 min, to account for potential persistence of impacts after the disturbance (e.g. [37]). Based on their Bayes information criterion [42], first-order Markov chains were preferred over zero-order chains.

For each behavioural indicator (group cohesion, school formation, aerial activity), we created 5-way contingency tables of preceding state, succeeding state, human activity or chain (Control/Pressure), location (Samadai/Satayah/Qubbat'Isa) and time of day (morning/midday/afternoon). Log-linear analyses [43] were used to test the effects of these factors on the behaviour transitions. Model fit started from a null model in which the probability of a succeeding behaviour state, given the preceding state, is independent of the other predictors; hierarchical models including, or excluding, specific effects were then fitted by maximum likelihood. Model selection was based on the Akaike information criterion (AIC, [43]), computed from the goodness-of-fit likelihood ratio statistic (*G*^2^) penalized by twice the degrees of freedom [44]. The best model minimized the AIC score, and models falling within 2 units were considered to have substantial support [45]. To investigate intra-site variability, the same procedure was repeated within location, including a field season effect (2006/2013/2014 for Samadai, 2013/2014 for Satayah, table 1 for definition of field season codes).

To describe changes in dolphin behaviour, we generated behavioural state transition matrices, under control and pressure conditions, for the morning, midday and afternoon time periods. We calculated stationary state distributions, independent of the starting distribution [43]*,* to quantify the proportion of time the dolphins spent in tight, inactive and single schools, states characteristic of resting behaviour [45–47]. We compared the behaviour state distributions under control and pressure conditions using a *z*-test for proportions [48], employing a Control–Impact inter-site approach (i.e. control state from the control site versus pressure state from tourism site for the same time of day) as well as an intra-site approach (i.e. control and pressure state from the same site, season and time of day). Directional Cohen's *h* was employed to describe the effect size of pressures, where *h* < 0.2 indicates a small, *h *∼ 0.5 a medium and *h *> 0.8 a large effect [46]. All analyses were carried out in the software package R [47]. Tests for proportion and Cohen's *h* used functions in the package *pwr* [49].

## 3. Results

Data were collected during 291 h of observation at Samadai (44 days in 2006, 2013 and 2014), 285 h at Satayah (29 days in 2013 and 2014) and 29 h at Qubbat'Isa (5 days in 2011). Spinner dolphin daily occurrence was high at all sites: encounters were recorded on 73% of survey days at Samadai, 93% at Satayah and 100% at Qubbat'Isa.

### 3.1. Human activities

Human activities were recorded on every day in which dolphins were present. Focal groups were exposed to boats and/or swimmers during 26% (±22% s.d.), 42% (±33% s.d.) and 53% (±23% s.d.) of their follows at Qubbat'Isa, Samadai and Satayah, respectively. Exposure was highest at midday at all sites (table 3).

**Table 3.** Focal groups exposure to interactions with research and tourism, and characteristics of the interactions per site (QI, Qubbat'Isa, SM, Samadai, ST, Satayah) and time category (MO, morning, MI, midday, AF, afternoon). TC, time category; *n*, number of focal group follows.

	exposure				pressure session			
			exposure	cumulative (h)	timing	duration (minutes)	median swimmer	median boat
site	TC	*n*	mean (s.d.)	mean (s.d.)	(min–max)	mean (s.d.)	mean (s.d.)	mean (s.d.)
QI	overall	11	0.26 (0.22)	1.9 (1.0) (*n* = 5 days)	09.50–17.20	42 (13.2)	0.08 (0.28)	1 (0)
	MO	2	0.00 (0.00)			0 (0.0)	0 (0.0)	0 (0.0)
	MI	5	0.33 (0.14)			48 (12.5)	0 (0.0)	1 (0.0)
	AF	4	0.31 (0.28)			34 (11.1)	0.17 (0.41)	1 (0.0)
SM	overall	90	0.42 (0.33)	3.1 (1.3) (*n* = 33 days)	05.40–16.00	73 (61.8)	11 (13.1)	0 (0.61)
	MO	25	0.40 (0.36)			41 (19.8)	2 (1.9)	0 (0.4)
	MI	35	0.66 (0.21)			85 (65.0)	13 (13.8)	1 (0.7)
	AF	30	0.15 (0.16)			24 (8.3)	2 (5.6)	1 (0.5)
ST	overall	71	0.53 (0.23)	4.9 (1.7) (*n* = 21 days)	05.15–17.15	75 (75.1)	4 (4.9)	1 (0.9)
	MO	24	0.44 (0.24)			65 (43.3)	3 (4.0)	1 (0.8)
	MI	24	0.62 (0.18)			102 (89.8)	6 (6.1)	2 (1.1)
	AF	23	0.51 (0.25)			54 (73.7)	2 (3.5)	1 (0.9)

At Qubbat'Isa, pressure sessions involved only the research inflatable and, occasionally, one researcher in the water, carrying out photo-identification data collection. They occurred in midday and afternoon hours and never lasted longer than 70 min (table 3). At Satayah, human activities were recorded as early as 05.00 and as late as 17.00, peaked at midday, and were sustained throughout the day, including the afternoon (table 3). At Samadai, they increased between morning and midday, to then decrease in the afternoon. At Samadai and Satayah, the two tourism sites, sessions had similar overall average duration (73 min and 75 min, respectively), were longer and involved more swimmers at midday (85 min with a median of 13 swimmers at Samadai, and 102 min with a median of six swimmers at Satayah) (table 3). At Satayah, the duration of sessions was longer and more variable than at Samadai in each time category (table 3). Overall, sessions at Samadai had more swimmers and fewer boats (table 3). Cumulative daily duration of human activity in the lagoons ranged 1–3 h at Qubbat'Isa, 0.5–5.7 h at Samadai and 2.1–8.9 h at Satayah, with average values of about 2, 3 and 5 h, respectively (table 3).

### 3.2. Dolphin short-term responses

We analysed 6204 behavioural transitions in cohesion (tight or loose), 6964 in school formation (single or multiple) and 7355 in aerial activity states (inactive or active). Log-linear analyses indicate that behavioural transitions in cohesion and aerial activity differed in the three sites in each time category (Time × Location effect), but that human pressures had the same time-specific effect at all sites (Chain effect) (table 4). The way schools transitioned between formation states was different among the study sites (Location effect), but was affected by pressures in a consistent way, regardless of the site and the time of the day (Chain effect) (table 4).

**Table 4.** Log-linear analyses of the effects of time, location and human pressure on behavioural transitions in cohesion, aerial activity and school formation. AIC, Akaike information criterion; ΔAIC, difference in AIC between best fitting and supported models. +, additive effect; *, interactive effect.

indicator	model	AIC	ΔAIC
cohesion	Time × Chain + Time × Location	15.30	
aerial activity	Time × Chain + Time × Location	0.45	
formation	Location + Chain	−36.96	1.81
	Location × Chain	−35.13	

At the tourism sites (Samadai and Satayah), transitions in cohesion and aerial activity differed among field seasons (Season and Time × Season effects) (table 5). Human pressure had consistent effects across seasons, with the exception of Samadai school formation (Season × Chain effect) (table 5). Pressures affected transition probabilities for all three behavioural indicators at Qubbat'Isa and Samadai, but only aerial activity at Satayah (Chain effect) (table 5). At Samadai, responses to pressures in cohesion and aerial activity changed with time of day (Time × Chain effect) (table 5).

**Table 5.** Log-linear analyses of the effects of time, location, field season and human pressure on transitions in cohesion, aerial activity and school formation. AIC, Akaike information criterion; ΔAIC, difference in AIC between best fitting and supported models. +, Additive effect; *, interactive effect; QI, Qubbat'Isa; SM, Samadai; ST, Satayah.

site	indicator	model	AIC	ΔAIC
QI	cohesion	Time + Chain	4.95	
		Time	5.48	0.53
	aerial activity	Time + Chain	−1.76	
		Time	−0.98	0.78
SM	cohesion	Time × Chain + Season	4.33	
		Time × Chain + Time × Season	4.41	0.08
	aerial activity	Time × Chain + Time × Season	−8.14	
		Time × Chain + Time × Season + Chain × Season	−7.37	0.77
	formation	Season × Chain	−16.61	
ST	cohesion	Time × Season	−11.65	
		Time × Season + Chain	−11.33	0.32
	aerial activity	Time × Season + Chain	−10.92	
		Time × Chain + Time × Season	−9.11	1.81
	formation	Null	−13.76	
		Time × Season	−13.61	0.15
		Time × Season + Chain	−12.78	0.98
		Pressure	−12.75	1.01

**Figure 3.**
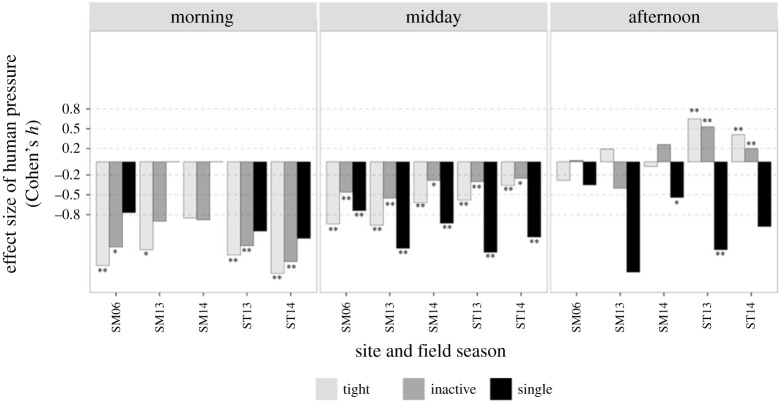
Time-specific effect of human pressure (inter-site approach): tight, inactive and single behaviour states (indicative of resting behaviour) at the tourism sites (SM = Samadai and ST = Satayah), compared to dolphin behaviour at the control site (QI = Qubbat'Isa), in the field seasons (06 = 2006, 13 = 2013, 14 = 2014). Values above zero indicate an increase in the occurrence of tight, inactive, single groups under pressure. Cohen's *h* ≤ 0.2 small, *h* ∼ 0.5 medium, *h* ≥ 0.8 large effect size. *p < 0.05, **p < 0.01.

When dolphins were exposed to pressures at the two tourism sites, there was a strong and consistent reduction in tight, inactive and single groups, indicative of resting behaviour, compared to control conditions at the control site (Qubbat'Isa), especially during the morning and midday periods (figure 3). There were some differences between field seasons in the strength of the response. For example, in the morning, Samadai groups were less responsive in 2013 and 2014 than in 2006, and than groups resting at Satayah (figure 3). Responses were more variable in the afternoon, when disturbances did not seem to affect Samadai groups, while they significantly increased the likelihood of finding Satayah schools divided into tight and inactive groups (figure 3).

**Figure 4.**
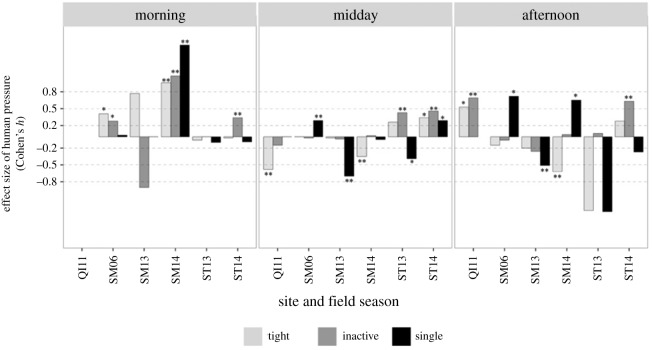
Time-specific effect of human pressure (intra-site approach): tight, inactive and single behaviour states (indicative of resting behaviour) from the same site, season and time of day. The alphanumeric codes on the *x*-axis indicate the site (QI = Qubbat'Isa, SM = Samadai, ST = Satayah) and field season (06 = 2006, 13 = 2013, 14 = 2014). Values above zero indicate an increase in the occurrence of tight, inactive, single groups under pressure. Cohen's *h* ≤ 0.2 small, *h* ∼ 0.5 medium, *h* ≥ 0.8 large effect size. *p < 0.05, **p < 0.01.

Intra-site analyses—contrasting behavioural data gathered in control and pressure conditions at the same site, season and time of day—showed that the direction and size of significant effects on cohesion and formation were highly variable among and within sites, field seasons and time of day (figure 4).

## 4. Discussion

Spinner dolphins in Egyptian waters regularly visit the three resting areas under investigation, and are exposed to tourism in the two more northern sites. Our assessment shows that human pressures at Samadai and Satayah reefs are organized in sessions of similar average duration. However, the time-area closure system at Samadai Reef ([30], figure 2) halved the daily cumulative duration of tourism interactions (on average), compared with Satayah Reef. The zoning system also substantially reduced the invasiveness and level of contact between dolphins and tourists compared with Satayah Reef. At Samadai, swimmers are prohibited to enter the dolphin-only area (Zone A) and powered vessels are only allowed in Zone C, thus away from the main area used by resting dolphins (Zone A) [30]. Moreover, at this site, while diving and navigation are allowed from 08.00 to 15.00, swimming and snorkelling are further restricted to the hours between 09.00 and 14.00, as per the management plan [30]. As a result, interactions at Samadai are concentrated in the central hours of the day, are less reliant on motorized vessels and involve swimmers who are spatially confined to their dedicated areas (Zones B and C). By contrast, dolphins at Satayah were sought after throughout the day, from dawn to dusk, and were observed being intensively and repeatedly approached by powered vessels and swimmers for up to 9 h a day.

The results of log-linear analyses showed that the tendency of dolphin groups to transition to, or maintain, states indicative of rest was sensitive to the presence and timing of human pressures. We found no support for site-specific effects of human interactions, indicating consistent dolphin responses to boats and swimmers at the three sites. Behavioural responses of Qubbat'Isa and Satayah dolphins were also consistent throughout the day. At Samadai Reef, there was more variation in dolphin responses to pressures depending on the time of day.

The comparison of control data from Qubbat'Isa with data collected in the presence of boats and swimmers in the tourism sites showed consistent disruption of dolphin rest at all times of the day. The proportion of time spent in tight, inactive and single schools was most strongly, and significantly, reduced during the central hours of the day. This is consistent with evidence from other studies that tourism operations cause increased surface aerial activity [50] and interrupt rest [23,31,51], especially during the middle of the day when dolphins should be in the deepest phase of rest [23,31]. Our results from Egypt indicate that dolphin groups break up in the presence of disturbances, rather than increasing coordination, as reported elsewhere [50]. At Satayah and Qubbat'Isa, groups appeared to respond to pressures also in the afternoon, a time of the day when the activity level of the school is normally heightened, as the school prepares to leave the resting area and travel to the foraging grounds [21]. In the afternoon, disturbances led to multiple groups, more likely to be tight and inactive, perhaps in an attempt to reduce energetic expenditure or minimize detection [52].

At Samadai Reef, we observed consistent responses to pressures during midday hours, but no significant response (i.e. habituation-type response) in the morning and afternoon. The amount of exposure estimated for the focal groups, as well as the daily cumulative values, were much lower at this site than at Satayah. In particular, Samadai dolphins were less often targeted by human activities in the morning and afternoon hours. Furthermore, the enforced zoning plan ensured a level of distance between the resting dolphins and human activities at this site, making interactions mainly initiated and pursued by the dolphins on their own accord (passive interaction) [1]. These factors make Samadai and Satayah two profoundly different systems and may drive the different responses at these two tourism sites. They could also play a role in the different morning responses recorded in the three Samadai field seasons: responses are stronger in 2006, when human pressure was higher and more invasive in the early morning hours, as research activities used to begin earlier than in 2013 and 2014, when they were effectively subjected to the same time and zone restrictions applied to tourism operations.

Research shows that spinner dolphins off Hawai'i rest within the confines of bays and lagoons and are unlikely to rest outside sheltered bays [25]. In Egypt, dolphin groups can find opportunities to rest in Samadai, where they can take advantage of the protected area (Zone A, figure 2) and times of day when tourism is not permitted [30]. By contrast, the close-up, invasive and intensive nature of interactions at Satayah, coupled with the significant acoustic disturbance that outboard engines can generate in the shallow depths of a bay [53], may render it difficult, if not impossible, for dolphins to rest in this resting area. The long-term site fidelity of the dolphin populations visiting this site [54] indicates that the individuals regularly occurring in Satayah are also repeatedly exposed to disturbances. If rest is confined to the lagoons, they are at high risk of chronic rest disruption. One possible adaptive response to achieve satisfactory rest in a suboptimal environment is the rearrangement of rest into shorter sleep cycles and more frequent awakening. This has been observed in animals after encounters with predators, sleeping in areas perceived to be less safe, or sleeping in smaller groups [55–57]. In the light of these considerations, we hypothesize that Satayah groups exposed to tourism disturbances over the last decade may abandon (or have abandoned already) the solid and long phase of rest, in favour of shorter rest bouts carried out in an opportunistic way, ‘taking a nap’ when conditions allow. The occurrence, quality, success and population-level effects of this and other adaptive responses are still unclear, but our findings raise considerable concern for the well-being of this population.

In order to accurately interpret behaviours observed, as well as to predict and assess population-level consequences, a dual approach that encompasses both the short- and long-term responses to human interactions is required. A recent study by Tyne *et al.* [19] concluded that regular, extensive, long-term use of resting bays as tourism sites has not resulted in abandonment of the bays, but rather in the decline of the dolphin population of Hawai'i. This suggests that resting areas are limited, and moving to another resting area is not an option. It is, therefore, important to consider that tourism impacts at resting areas may result in reduced dolphin reproduction and/or survival. Using baseline data on the Samadai and Satayah population size and site fidelity [54,58] to establish a long-term programme to monitor population trends and dynamics is a high priority. The Egyptian case study benefits from conditions particularly suitable for investigations of behaviour and trends in abundance over time, because it focuses on small, resident and easily accessible dolphin populations [59]. This study, the first to investigate the effects of human interactions in Egypt, should be advanced with efforts to better understand the species' ecology and organization on a regional scale and outside the resting areas considered. For instance, habitat modelling could help identify potential additional resting areas and be followed by field and photographic identification surveys. Moreover, as Egyptian spinner dolphins often occur offshore in association with pantropical spotted dolphins (*Stenella attenuata*) [60]—the mixed assemblages possibly providing alternative favourable resting conditions for the spinner dolphins [61]—the individual composition of these offshore groups could reveal information on resting strategies alternative or complementary to the use of resting areas.

While long-term and site-specific studies of population distribution, abundance and survival are needed to assess the population-level impacts of human disturbance on this spinner dolphin population, the findings presented here clearly demonstrate that welfare of individual dolphins resting at Satayah Reef is being affected. As recently emphasized by Papastavrou *et al.* [62] approaches that include both conservation (i.e. population decline) and welfare (i.e. individual well-being) of marine mammal populations can lead to more effective and efficient management decisions involving wild populations [62]. This combined conservation–welfare approach supports immediate actions in Satayah and ongoing validation of the Samadai management plan at the individual and population level.

Spinner dolphins in Egypt provided an excellent study system for the investigation of dolphin population ecology and behavioural responses to tourism impacts. The dataset from Qubbat'Isa, the control site, was small, due to the difficulty of gaining access to this military zone. However, data on spinner dolphin behaviour in the absence of human activity were extraordinarily valuable in assessing responses to human interactions in the two tourism sites. In particular, the comparison with the control site was essential to confirm that none of the data collected at Samadai and Satayah reefs could be considered ‘control’ data, unaffected by tourism. The frequent exposure to tourism at these sites may not allow dolphins to return to truly undisturbed behaviour and this can make it difficult (if not impossible) to detect differences between dolphin behaviour in the presence and absence of tourism, as it occurred when we applied an intra-site approach.

Our results open numerous avenues for future research to test, confirm, complement and expand current knowledge on spinner dolphins. Further research would help to increase sample size and data on additional indicators of rest (e.g. vocalization [50,63] and movement patterns [64]), as well as on group composition (e.g. age, sex, activity state [14,24]) and broader ecological and social factors (e.g. previous exposure, dynamics within the population [65]) from all sites. Recent studies on spinner dolphins suggest that the number of vessels or tourists alone is not a sufficient predictor of responses [54,66]. Indeed, proximity, invasiveness, duration and type of interactions are important features to consider [63,67]. These variables, however, were very difficult to control in observations of dolphin interactions, due to the numbers of boats and swimmers constantly changing during the pressure sessions. Also, while our study focused on close-up interactions, it cannot be excluded that activities occurring within a larger range from the focal group could have had an effect. Our methodological choices could also have affected our results: Meissner *et al.* [68] found that decisions on the allocation of samples to pressure or control chain affected the predicted values returned by the models. Future studies should address the uncertainty pertaining to samples at the beginning and end of interactions, ideally by defining multiple stages within the interaction (as preliminarily attempted in [54]) or resorting to other analytical frameworks, such as time series. As new data collection techniques become available, their suitability for tourism impact assessment studies should also be evaluated. For instance, the use of unmanned aerial vehicles (UAVs) for the collection of information on tourist operations and dolphin groups (e.g. number, location, relative position, distance) over a larger area should be investigated. Recent technological and methodological developments also include the analysis of metabolite content of cetacean blows, proposed as an effective and non-invasive way to assess individual stress levels [69]. We hope that opportunities to apply it to wild small cetaceans will soon be developed because information on physiological responses, undetected in behavioural studies [70], could provide quantitative and robust indicators to predict and interpret individual and population reproductive and growth rates. In conclusion, we recommend continued investigation of the effects of research and tourist operations to address some of the above data gaps and to incorporate new data collection methods. The Egyptian natural experimental setting is ideal for designing additional studies on the effects of different human activities and supporting sustainable management of whale watching operations.

## 5. Conclusion

Our study emphasizes the importance of interpreting observed behaviours in conjunction with historical and contextual information. The lack of a control site might have led us to underestimate the behavioural responses of dolphins to tourism, reach inappropriate conclusions on their conservation status and formulate inadequate recommendations for tourism management. We believe that progress towards sustainable management of tourism operations targeting wild dolphins requires the adoption of a precautionary approach, taking note of the conclusions and lessons learned in the best-documented sites [71]. This is essential when tourism targets small, closed and resident communities of cetaceans [71], and for populations or species, such as the spinner dolphin, that rely on specific locations for activities as critical as resting. Such conditions generate severe concerns for both the welfare and the conservation of the population. We support current claims that both aspects are worth consideration for the formulation and assessment of management plans [62], a view that is emerging in intergovernmental organizations such as the International Whaling Commission [72]. Supporting the position of international institutions, we urge Egyptian authorities to strictly regulate existing operations in Egyptian resting areas [8] and advise against the further development of swim-with dolphin tourism [73]. Any unregulated increase in dolphin tourism should be discouraged. We considered the Samadai management model to be inspirational, as it substantially reduced the cumulative exposure of the dolphins to human interactions; accordingly, site-specific regulations (such as time-area closure) should be devised elsewhere, where appropriate, to protect the species' most critical spaces and ensure resting opportunities. In particular, management interventions are urgently required at Satayah Reef. Since the management of wildlife-oriented recreation lies at the juncture of the biological and social sciences [74], we envisage that progress made in the natural sciences will be paralleled with advances in the study of the socio-economical, cultural and governance aspects of this tourism industry for the formulation of most effective and successful schemes.

## Supplementary Material

Details on Materials and Methods

## Data accessibility

The datasets supporting this article are available on M.F. Open Science Framework account at (https://osf.io/3sgde) [75].
