# Supplementary material for: Behavioural responses of spinner dolphins to human interactions
Source: R Soc Open Sci. 2018 Apr 25;5(4):172044. doi: 10.1098/rsos.172044 (PMC5936925; doi:10.1098/rsos.172044)
Supplement: Details on Materials and Methods [file rsos172044supp1.docx]

ESM1

**Details on Materials and Methods**

Additional information on the study surveys and data standardisation protocols.

**Surveys organisation**

The 2006 survey was carried out within the 1-year “Abu Salama Project” funded by the Italian Cooperation in Egypt. The survey included photo-identification and behaviour data collection. No further surveys took place until the design of a second project was completed, its approval formalised and funding released. This second project, the 3-year long “Sustainable Development in the Southern Egyptian Red Sea” project, again funded by the Italian Cooperation in Egypt, was launched in 2010. The 2011 survey in Qubbat’Isa occurred within one of its component, the “Red Sea Dolphin Project”, a regional programme for the investigation of cetacean abundance and distribution in the Egyptian coastal waters south of Marsa Alam. Due to research aims and logistical issues, in these years surveys in Samadai and Satayah were mainly focussed on the collection on photo-identification data. The 2013-2014 surveys in Samadai and Satayah included both behaviour and photo-identification data collection and were organised as part of the doctoral studies of MF (spinner dolphin behavioural responses to interactions) and AC (Samadai population ecology).

**Sampling regime**

The 2011 survey employed a number of sampling regimes in the attempt to optimize the data collection. These included 60-second and 120-second intervals and, for groups displaying synchronous breathing patterns, observations at each group surfacing. These regimes resulted impractical (e.g. samples too close in time to collect all the variables) [54], therefore 2013 and 2014 seasons employed the original 150-second regime.

In order to avoid biases due to over- or under-sampling, data from the 2011 survey were interpolated to 150-second intervals. In order to do so, the behaviour was assumed constant between the closest original sample and the interpolated sample. If no original sample was available within one minute from the interpolated time, the sample was left blank. For example, data for the interpolated time 10:25:00 were searched among original samples collected between 10:24:00 and 10:26:00. Among the valid samples, the closest to the interpolated time (either preceding or succeeding) was selected as the most representative. The behaviour was assumed invariant and the data from the original sample were attributed to the new sample. If no samples were available within one minute, the standardised sample was considered missing and left blank. All subsequent analyses were carried out on 150-second sampling interval datasets.
